# New Directions in Environmental Justice Research at the U.S. Environmental Protection Agency: Incorporating Recognitional and Capabilities Justice Through Health Impact Assessments

**DOI:** 10.1089/env.2021.0019

**Published:** 2021-10-04

**Authors:** Emily Eisenhauer, Kathleen C. Williams, Camilla Warren, Tami Thomas-Burton, Susan Julius, Andrew M. Geller

**Affiliations:** Office of Research and Development, U.S. Environmental Protection Agency, Washington, District of Columbia, USA.; Office of Research and Development, U.S. Environmental Protection Agency, Duluth, Minnesota, USA.; U.S. Environmental Protection Agency, Atlanta, Georgia, USA.; U.S. Environmental Protection Agency, Atlanta, Georgia, USA.; Office of Research and Development, U.S. Environmental Protection Agency, Washington, District of Columbia, USA.; Office of Research and Development, U.S. Environmental Protection Agency, Durham, North Carolina, USA.

## INTRODUCTION

The U.S. Environmental Protection Agency’s (EPA) mission is to protect human health and the environment for all people and communities in the United States, guided by environmental science. However, the traditional scientific approaches that support the agency’s work, such as risk analysis and assessment, have well-known limitations for advancing Environmental Justice (EJ).^1,2^ The dependence upon particular kinds of data analysis can lead to delays, failure to consider multiple and cumulative stressors and underlying conditions, limited choices for remediation, and reinforcement of existing power relationships without engaging communities.^3^

As a result, communities and tribes often report feeling like objects of research rather than collaborators,^4^ having little control over research or how data developed within their communities were used and reported and too often finding few material benefits from the research.^5^ However, using research approaches that encourage new and more democratic ways of doing science brings community members into the process and provides a way for them to be active contributors to research that informs community solutions. Such approaches are showing significant potential to address EJ issues.^6^

Residents of the Proctor Creek watershed, a 16 square mile area between downtown Atlanta and the Chattahoochee River,^7^ have long raised concerns about contamination of the creek. As early as 1901, Black leaders protested sewage releases in the creek, leading the city to install one of the first sewage plants.^8^ By the early 2000s, extensive development and impervious surfaces over the headwaters of the creek frequently resulted in downstream flooding and combined sewer overflows (CSOs) that caused the creek to overflow its banks and sewers to back up into homes.^9^ Neighborhood organizations and community members have conducted extensive community research and citizen science documenting these conditions, including sewage releases, illegal dumping, mosquitoes, and unhealthy housing conditions due to inundation from flash flooding and compromised water quality.^10^

Despite this long history of activism, Proctor Creek is one of many examples^11^ where environmental injustices have persisted, with resolutions delayed by lack of recognition and the limitations of formal regulatory processes. EPA’s definition of EJ calls for “fair treatment and meaningful involvement” in the process of environmental protection,^12^ but numerous institutional, legal, cultural, and individual barriers have hindered the realization of this vision.^13^ Indeed, fair and meaningful may be contested terms, understood differently by residents and city officials or other experts^14^ and not seen as meaningful or as having a satisfactory impact on outcomes.^15^ Given this array of limitations, scholars now argue that meaningful EJ work requires a deeper theoretical foundation for EJ that goes beyond distributional and procedural justice and includes attention to recognition and capabilities.^16^ Recognitional justice is based on the understanding that failure to acknowledge the lifeways, culture, and values of those affected by environmental problems devalues individuals and communities, thereby allowing injustice to exist.^17^ Capabilities justice acknowledges that equity cannot be achieved without attention to the contexts in which people live, and to the provision of goods and opportunities for people to participate in governing processes and that allow them to live the life they chose.^18^ Distributional, procedural, recognitional, and capabilities justice are intertwined; without recognition and capabilities, fair distribution and meaningful involvement cannot be achieved.^19^

In this commentary, we provide an example of how a broader conceptualization of EJ that includes these four dimensions can elevate community voices to achieve more just outcomes. In Proctor Creek, although the EPA Region 4 Office was supporting local efforts to return the creek to Clean Water Act compliance through technical assistance and small grants, residents felt that their concerns about the impacts of flooding and contamination were not being adequately addressed and asked EPA to become more involved in addressing these EJ issues. Recognizing that the communities in the watershed were experiencing environmental injustice, the EPA Regional Office began a concerted effort to engage and build long-term relationships with the communities in the watershed, which brought additional attention and resources to the concerns of residents. As part of this engagement, EPA initiated a Health Impact Assessment (HIA) to study how implementing Green Infrastructure (GI) in the watershed could address their EJ concerns.^20^ This assessment process provided a path for meaningful engagement among the community, stakeholders, and EPA through the recognition of community concerns, a procedure to facilitate engagement with citizens and agencies, and a fairer distribution of environmental goods through the addition of GI. By using health as an integrating concept and HIA as a methodology, community and government stakeholders were able to take concrete steps to address the interrelated issues of poor water quality, flooding, environmental degradation, public health impacts, and lack of economic opportunity, enabling them to be considered together while implementing GI projects, thereby improving community capabilities. In this commentary, we demonstrate the promise of HIA’s and community engaged approaches generally as a way for EPA to address EJ issues, and argue for an expanded approach to EJ that incorporates these four dimensions to create the conditions for collaborative solutions that have material impact on the human health and environment in our communities.

## THEORETICAL DEVELOPMENTS IN EJ RESEARCH AND PRACTICE

While early EJ research on the distribution of exposures and impacts was critical to advancing the emerging EJ movement,^21^ the field has broadened to include issues of access to environmental amenities, long-term community resilience, and promotion of health and well-being,^22^ and to incorporate additional dimensions of justice, namely recognitional justice and the capabilities approach.^23^ Recognitional justice ensures individuals’ and communities’ claims for participation in decision making affecting them are acknowledged and enacted.^24^ Recognition of one’s culture, identity, and place in society underlies one’s ability to participate as a full member of society, and thus, misrecognition hinders both procedural and distributional justice. EJ researchers are demonstrating how the lens of recognition can introduce specific knowledge related to a community’s culture, history, or economic situation, which may be left out of risk analysis, a technical scientific process to identify acceptable amounts, toxicity, and exposure potential for a chemical.^25^ For example, Holifield^26^ traced the ways that the Leech Lake Band of Ojibwe’s (LLBO) struggle for recognition of their tribal territory and culture played a role in a risk assessment process at a Superfund site. EPA’s failure to adequately recognize the tribe, their culture, and relationship to the land in the original research led to potentially greater risk of exposure for tribal members. The LLBO’s demands for recognition led to greater consideration of their traditional tribal lifeways and local knowledge in the risk assessment process, and, in turn, led to results that were more appropriate for their interactions with the environment than are the national standards that are based on more suburban lifeways. The LLBO example illustrates how the complicated relationship between indigenous groups and the U.S. government, still largely based on colonial ways of knowing and being, means that only partial recognition may be possible and shows how lifeways and experiences can be rendered invisible unless tribes affirmatively assert their rights.

The capabilities approach to justice grew out of the human development work of Amartya Sen^27^ and Martha Nussbaum^28^ and “focuses on the specific range of basic needs and capabilities (including recognition) that human beings require to function.”^29^ For Nussbaum, capabilities are the opportunities for functioning and choice that are available to people, and which is each person’s human right, and the capabilities approach is “concerned with whether people are able to translate those protections and goods into actual achievements that characterize a life that is worthy of the dignity of human beings (p. 321).”^30^ Examples of capabilities are economic and social relations and resources, physical and mental health, emotional resources, and control over one’s environment. Capabilities are the “real freedoms” that are “not merely the formal freedom to do or be something, but the substantial opportunity to achieve it.”^31^ For Sen, functioning is actualized through choice,^32^ which in the context of governing requires additional consideration to ensure that agencies facilitate public input processes that are transparent, genuinely consider input, and are not perfunctory. Capabilities, distribution, recognition, and procedural justice are interconnected; scholars argue that it may be impossible to achieve justice of capabilities unless there is intentional focus on distributional justice, which requires some type of redistribution of environmental “bads” and “goods”. It may aslo be impossible to achieve such redistribution without recognition and meaningful procedure.^33^

Public engagement procedures by governmental agencies, including EPA, are formal meetings, often held toward the end of the research or decision and organized to fulfill agency requirements by streamlining the collection of public input.^34^ This type of formal process reflects structural inequalities in society, discouraging participation by community members who do not feel comfortable in those settings or feel their input will not impact any change.^35^ Recommended improvements to these processes, such as those developed by the National Environmental Justice Advisory Committee,^36^ incorporate principles from recognitional and capabilities justice as well as procedural justice to make community engagement processes more meaningful and effective. Research by environmental agencies can also facilitate new modes of engagement that serve both communities and the agency. EJ can be advanced by conducting research that demonstrates the ways in which communities are misrecognized or denied the necessary capabilities, and incorporates recognitional and capabilities justice in the research process.^37^ While EPA has supported community engaged research externally for some time,^38^ it has more recently begun to build greater capacity for community engaged approaches through partnerships between the EPA ORD and EPA Regional offices, which administer EPA programs and policies with their states and lead agency efforts to address environmental issues at the community level. Through these efforts, research is playing a role in addressing EJ issues that goes beyond traditional distributional and procedural justice approaches.

One approach that holds significant promise for incorporating recognitional and capabilities justice into EPA’s community engagement processes is Health Impact Assessment (HIA). HIA features significant stakeholder engagement and community participation at the beginning and throughout the process as part of the methodology and is based on core values of democracy and health equity. HIAs assess how social determinants of health, which are related to capabilities in that they call attention to factors underlying health and well-being, are impacted by policy changes.^39^ HIAs that analyze impacts on environmental decisions further address environmental decisions as a “meta-capability,” which Holland^40^ argues are instrumental to all other capabilities in supporting the health of individuals and communities.

EPA has worked with community and government partners to evaluate and pilot HIAs in the context of local decision making around flooding and sanitary sewer issues, school renovation, and site remediation^41^ to determine whether community-engaged equitable processes yield decision outcomes that result in long-lasting benefits to the community. In the following section, we provide an example of HIA and Story Mapping conducted in the Proctor Creek watershed in Atlanta, Georgia, as an example of how research and science-based decision processes can incorporate recognitional and capabilities justice, in addition to furthering distributional and procedural justice.

## RECOGNITIONAL AND CAPABILITIES JUSTICE IN HIA AND STORY MAPPING IN THE PROCTOR CREEK WATERSHED

In this section, we first describe how the HIA and Story Map unfolded and then follow with a discussion of how these activities incorporated the four dimensions of justice, and the impacts and limitations of this approach.

A thriving area in the first half of the twentieth century, environmental and economic conditions in the Proctor Creek watershed have declined since midcentury due to disinvestment and population decline driven by urban renewal and suburbanization, with several neighborhoods in the watershed experiencing high poverty, unemployment, vacancy, and crime rates.^42^ Although within walking distance of downtown Atlanta, physical barriers such as a highway and convention complex inhibit connectivity, and issues related to the creek such as flooding, dumping, and contamination are significant barriers to economic activity. Community members and community-based organizations (CBOs) such as the West Atlanta Watershed Alliance, Eco-Action, and neighborhood groups had been active for some time in promoting better environmental conditions through water sampling and cleanup activities, educational programs, and advocacy with the city of Atlanta.^43^ Interest among these groups and the city in GI as part of the solution to the problems stemming from the degraded condition of the creek was growing, and in 2009, neighborhood residents reached out to a local nonprofit organization Park Pride to investigate GI solutions to stormwater issues. Park Pride led a coalition of neighborhood residents and organizations in identifying flood-prone areas and developing a plan for implementing GI and increasing green space in the watershed, culminating in a major report, the 128-page Proctor Creek North Avenue Watershed Basin study.^44^

The city identified funding to implement a demonstration project from the plan, the “Boone Boulevard Complete Streets Project,” to address water quality issues and relieve the burden on the existing inadequate stormwater infrastructure. Community and government stakeholders recognized that the issues of poor water quality, flooding, environmental degradation, public health impacts, and lack of economic opportunity were interrelated and needed to be considered together in implementing GI projects to adequately address EJ concerns. EPA, as part of its efforts to expand engagement with community stakeholders and support their efforts, proposed conducting an HIA. The HIA would elevate community concerns and integrate these concerns and community knowledge around health issues with scientific knowledge to provide evidence-based recommendations to the city for addressing the problems with the creek and its impacts in the neighborhoods. Discussions with community leaders, the city, and other stakeholders determined that there was sufficient interest and willingness to participate in the HIA, and the city provided information on specific points in the project planning process where information from the HIA could inform decisions.

HIA is defined by the National Research Council as “a systematic process that uses an array of data sources and analytical methods and considers input from stakeholders to determine the potential effects of a proposed policy, plan, program or project on the health of a population and the distribution of those impacts within the population (NRC 2011).” The HIA methodology begins with collaborative problem formulation based on the social determinants of health and uses an iterative approach with community engagement throughout the process. It includes building an evidence base to assess the potential impacts of different development options on community- and other stakeholder-defined objectives, especially health impacts. HIA follows a rigorous six-step process: (1) screening, (2) scoping, (3) assessment, (4) recommendations, (5) reporting, and (6) monitoring and evaluation. The process involves working with a team of community members stakeholders to identify potential health risks and benefits of a proposed policy, program, or plan, such as the Boone Boulevard project, from the earliest part of the process to then guide its implementation and maximize positive impacts while minimizing negative ones.

The Boone Blvd Project HIA was led by Region 4 and EPA’s Office of Research and Development with a core team responsible for the heavy lifting of data collection, analysis, and project management. The core team worked closely with a group of CBO representatives and other community members and leaders, and an advisory group that provided technical expertise and local knowledge and included representatives from multiple EPA Region 4 divisions, CBOs, City and County departments, six federal agencies in the Urban Waters Federal Partnership (UWFP), universities, and the state of Georgia (Table 1). During the conceptual design stage with the city, points were identified in the planning process where the HIA could influence decision making. The process included gathering community input on key concerns, conducting a literature review, and analyzing quantitative and qualitative data to characterize and rate potential impacts of decision alternatives on the strength of the evidence. Community input was used during the scoping phase to identify 12 health determinants for study, including water and air quality, flood management, economy and jobs, social capital, access to green space, and health care, and to refine and score final recommendations.

After the HIA report was released in 2016, community members expressed the desire to have a format that would make information about health concerns and GI projects for the entire watershed more easily accessible. EPA worked with a group of CBO and community leaders to produce a Story Map,^45^ an online platform for displaying maps and textual information, that provides information about additional GI projects being implemented throughout the watershed and displays data on watershed areas most likely to experience flooding, heat impacts, and mosquito infestations, noting those as locations where future GI projects could have the greatest benefit for the community. The Story Map is a coproduced resource that makes the HIA findings more widely available than a standard report document because it is highly visual and interactive. It provides additional resources to support inclusion of residents’ health and quality-of-life concerns in GI project planning implementation in the watershed, although it does not include project-specific recommendations.

The HIA findings and recommendations were intended to be applicable to the Boone Blvd Project as well as support further GI projects in the watershed. The Boone Blvd Complete Streets project was expanded by the city of Atlanta into a more robust GI program, portions of which were funded by private foundations and the first ever publicly offered Environmental Impact Bond.^46^ Through the efforts of the partners convened for the HIA, the watershed is now home to 21 completed or planned GI projects, both publicly and privately funded (EPA 2020). The Army Corps of Engineers completed an Ecosystem Restoration Study^47^ as part of an agreement with the city of Atlanta that included working with community members, CBO leaders, and the UWFP. As a result of the ongoing community and CBO “Parks with Purpose” initiative,^48^ two parks were created with GI features, one that, at the request of community members, included an underground cistern for holding potentially contaminated stormwater to further reduce flooding risk. The parks provide recreation and social space, and local residents were trained and employed in construction of the parks. These parks and other GI projects contribute to the equitable distribution of environmental amenities, including ecosystem services such as flood control, green space, and clean waterways in Atlanta.

## DISCUSSION

Agyeman *et al*. note that “the concepts and practices of EJ are open to, and encompass, varied notions of justice as they apply to given contexts and concerns.”^49^ We would contend that the HIA was an open and transparent process, where input from the community was recognized and meaningfully considered—reflecting both recognitional justice and capabilities in addition to enhancing the distributional and procedural justice aspects. Table 2 illustrates how the Boone Boulevard Project HIA goals reflect each dimension of justice. Goal 1 reflected procedural and recognitional justice by actively involving those who are directly impacted in decision making, going beyond simple public comment, and legitimizing their observations and experiences through research. Goal 2 addressed distributional justice and justice of capabilities by emphasizing the provision of beneficial infrastructure in underserved neighborhoods and by incorporating consideration of economic and societal impacts into the process. Goal 3 addressed distributional justice and capabilities, emphasizing the provision of services to the community by the built and natural environment (metacapabilities) that were not previously provided. Goal 4 addressed justice of capabilities, emphasizing the aspects of community empowerment, ownership, and accountability that are needed to address systemic inequities.^50^

The HIA reflected recognitional justice by including community members and organizations early on during the scoping phase where the most important determinants of health to assess are identified, and throughout the process. This places the community front and center in the research problem formulation and helps ensure that their input and participation are treated as equal with others such as government agencies, academic scientists, and planners. Any community input submitted throughout the HIA process is treated the same as peer review comments that shape the scope, assessment, and recommendations, and must be addressed.^51^ Participation during the scoping step recognizes community priorities and addresses concerns about how the planning action or policy change might impact them, ensuring their concerns are integrated with scientific research into cohesive findings and recommendations. The collaboratively produced HIA report provided a transparent record of the HIA recommendation process, including how community feedback was incorporated, exemplifying procedural justice (Table 3).

One of the major ways that the HIA enhanced the community’s capabilities was by ensuring that the community’s experiences and knowledge were considered at the same time as the technical studies through a research product. It also connected community members to agencies and resources, building a network to expand community capacity and sustainability, thus addressing justice of capabilities. The overlapping stressors of flooding, CSOs, mosquitoes, illegal dumping, derelict housing, mold, and asthma could only be addressed by multiple federal, state, and local agencies and CBOs working collaboratively. By convening this broad stakeholder group, the HIA supported the development of a collective vision for how to address the many problems, and the Story Map made the resources identified by the HIA available to the community at large, supporting ongoing work to address these issues. While this does not necessarily guarantee that community members have equal say in final decisions that are made by various agencies, the increased attention and marshalling of resources have made a substantial difference, and specific community knowledge has influenced the implemented projects on a number of occasions, such as the installation of the underground cistern at Katherine Johnson Memorial Park included in the design at the request of community residents for whom reducing flooding was of highest priority.

The HIA and the Story Map also reflect recognitional and capabilities justice by centering residents’ concerns of health and quality-of-life issues in the presentation of scientific findings, using a form that is more broadly useful and durable beyond the Boone Boulevard project. They describe the connections between environmental issues and community-defined health objectives, increasing the knowledge capacity of community members, stakeholders, and decision makers. The Story Map shares evidence from the HIA in a way that is accessible to the community and recognizes the key leadership roles that residents and CBOs have taken to address the multiple stressors related to the creek. Importantly, developing and publicly releasing the Story Map increase the likelihood that community concerns around health issues associated with Proctor Creek would continue to be addressed in planning and implementing GI projects after the HIA was completed, reflecting common concerns about research that does not result in concrete improvements for communities.

### Limitations

While these research products clearly reflect aspects of recognitional and capabilities justice, they did so without explicitly defining these concepts, thus missing the opportunity to fully realize them. First, the HIA report did not include an assessment of neighborhood culture or history, and so, potential cultural or social impacts and recommendations for addressing them may be missing, compromising full recognition. For example, gentrification was discussed as a potential impact only in the economic sense (e.g., displacing lower income households) and not in the sense of the potential social or cultural impacts (e.g., of changing racial and ethnic demographics through displacement). Second, although the report avoids stereotypes and other forms of misrecognition through the great care taken to be transparent in how conclusions were drawn based on data, it does not include assets of the community that are based in culture and social connections, which could have illustrated existing capabilities and agency for new efforts to build upon. The Story Map fills this gap to some extent by adding context on the role that community residents and nonprofit organizations played in improvements in the watershed, specifically mentioning some grassroots community organizing efforts, and linking to other sources of more extensive community-oriented resources. Accounting for cultural, social, and historical dynamics in assessments such as HIAs is important for ensuring that recommendations are culturally and socially appropriate, and may foster recognition to help alleviate mistrust, reducing the potential pushback from residents that GI projects sometimes encounter and ensuring residents benefit from the capabilities that the GI project would bring.^52^

The advantages of HIAs for government agencies such as EPA are that they are highly structured, but also transparent. However, HIA does not “fix” some of the challenges faced by agencies that are actively working to address environmental problems, such as navigating responsibilities to multiple parties. One challenge is that local decision makers may not be willing to participate as stakeholders in the process and are under no obligation to take HIA recommendations. Stakeholders and communities often choose to participate because the process ensures that all concerns are considered throughout, increasing the likelihood that recommendations will be adopted. Similarly, monitoring and evaluation require ongoing community and stakeholder commitment. The core team conducted a process evaluation that confirmed that the HIA had met its four goals, and indicators and data sources for monitoring were included in the report, although the report did not assess feasibility of monitoring. A number of subsequent activities such as water quality monitoring by the EPA^53^ and CBOs, community-led participatory research on environmental stressors,^54^ and continued advocacy by community organizations and stakeholders have ensured that the HIA results continue to inform decisions impacting conditions in the watershed.

Another challenge is building trust, relationships, and capacity for engaging in decision processes so that community members and CBOs can participate fully. In the Boone Boulevard HIA, stakeholders received training on the HIA method that helped build understanding and support for the process. Before the HIA, EPA and the UWFP recognized the key role of local CBOs such as WAWA and the Proctor Creek Stewardship Council (PCSC) in leading efforts to address EJ issues related to the creek, and provided several grants for expanded engagement in the watershed. The grants supported a series of community roundtables that highlighted a broad range of community concerns and led to the further engagement of other federal-, city-, and faith-based partners to address these concerns; in the process, building the capacity of local organizations such as the PCSC, which was able to incorporate as a 501c3 organization. This increased capacity and network of partnerships have strengthened community-led efforts, and CBOs have begun to investigate additional environmental issues such as mold and soil contamination.^55^ The EPA Region 4 Office continues to support these efforts with technical assistance as resources permit.

## CONCLUSION

This short description of the purposes and activities of an HIA touches on how multiple dimensions of justice can be integrated in EPA research. In attempting to address the unequal distribution of benefits and burdens by recognizing the environmental injustices faced by communities in the Proctor Creek watershed, the Boone Boulevard HIA and Story Map project addressed distributional justice; by identifying and involving residents of the watershed and their longtime concerns, they used recognitional justice; by enabling meaningful involvement, they strove for procedural justice; by helping build the capacity of the communities and creating opportunities to affect their desired changes, they increased capabilities justice.

The Boone Blvd HIA and Proctor Creek Story Map demonstrated that an HIA conducted as part of a broader EPA Regional and multistakeholder effort has much greater potential for addressing these four dimensions of justice—distributional, procedural, recognitional, and capabilities—than the traditional approaches to environmental regulation alone. Providing resources and developing methods that enhance community participation and utilizing knowledge and connections in the community are key to the success of an HIA. As an example of community-engaged research, this HIA included community voices early on to inform assessment and option selection, created opportunities for convening a wide array of stakeholders, and provided a method for considering interrelated water quality, economic, and health issues in the watershed, which supported the goals of community members, the EPA, the city of Atlanta, and numerous other stakeholders.

Addressing environmental injustice in communities requires pursuing justice broadly defined. Applying recognitional justice would mean recognizing community members’ claims about how injustices impact their lives and limit their capabilities. Justice of capabilities would call for policies, procedures, and methodologies to recognize and enhance capabilities to increase community well-being. As researchers at EPA, we can begin to create a greater awareness of how researchers and programs can identify opportunities for community collaboration in their own work. This type of research will allow EPA to be more intentional about applying these dimensions of justice in future research and other HIAs. For example, through an HIA, EPA could solicit what the parks and neighborhoods mean to community members to better characterize the capabilities they value, broadening EPA’s knowledge of communities beyond their stressors and creating space to talk about the cultural and historical dimensions of their place.

Recognition and capabilities strengthen translational research, which requires involvement by stakeholders to shape the research and ensure that real-world impacts are realized. Explicitly applying the concepts of recognitional and capabilities justice would enhance EPA’s use of HIAs, Story Maps, and other products that center on community concerns and incorporate their social and cultural aspects to provide a deeper understanding of injustice and additional ways to address it. Drawing on a capabilities approach to EJ would enhance efforts to address the distribution of environmental hazards. As the example of Proctor Creek shows, by recognizing community-led efforts and supporting the development of networks and organizational structures in the community, the capacity for environmental protection was built, supporting community well-being into the future.

## Figures and Tables

**Table 1.** Stakeholder Participation in the Boone Boulevard Health Impact Assessment

HIA core project team	
EPA Region 4, EPA ORD, U.S. Centers for Disease Control, WAWA, Georgia State University, Fulton County Department of Health and Wellness	
HIA advisory group	
Federal Agencies	CDC, HUD, USDA Forest Service, FEMA Region IV, Federal Highway Administration
State Agencies	Georgia Department of Transportation, Georgia Department of Public Health, Georgia Environmental Protection Division
Local Public Agencies	City of Atlanta Deparment of Planning and Community Development, City of Atlanta Neighborhood Planning Unit, Atlanta Regional Commission, City of Atlanta Department of Waste Management, Invest Atlanta
NGOs	The Conservation Fund, WAWA, Atlanta Beltline, Inc., Trust for Public Lands, Environmental Community Action, Inc., English Avenue Neighborhood Association, Park Pride, Community Improvement Association, Inc., Chattahoochee Riverkeeper
Academic Institutions	Georgia State University, Georgia Tech, Kennesaw State University, Spelman University, Emory University

CDC, Centers for Disease Control; EPA, Environmental Protection Agency; FEMA, Federal Emergency Management Agency; HIA, Health Impact Assessment; HUD, U.S. Department of Housing and Urban Development; USDA, U.S. Department of Agriculture; WAWA, West Atlanta Watershed Alliance.

**Table 2.** Boone Boulevard Health Impact Assessment Goals

Boone Boulevard HIA goals
Goal 1: Add a vehicle for equitable inclusion of all stakeholders in the decision-making process. Goal 2: Assess the effectiveness of the proposed GI project and raise awareness of the environmental, economic, and societal impacts of implementing GI in the designated community. Goal 3: Provide recommendations to the proposed project that incorporate approaches to stormwater management, ecosystem restoration, and community revitalization. Goal 4: Increase transparency, local accountability, community empowerment, and ownership of the proposed plan through meaningful stakeholder engagement.

GI, Green Infrastructure.

**Table 3.** Process Steps and Environmental Justice Dimensions in the Boone Boulevard Health Impact Assessment

HIA process step	Considerations	Dimensions of justice
Screening	Determined if there were community health concerns that should be considered in relation to the project and if an HIA could provide useful information to the decision-making process	Recognitional, procedural
Scoping	Identified scope of impacts (geography and populations); established project plan and stakeholder involvement; gathered community input on health concerns and modeled pathways to proposed project	Recognitional, procedural
Assessment	Described baseline status of health determinants; collected evidence from data and scientific literature to forecast potential impacts	Recognitional, procedural, capabilities justice
Recommendations	Identified strategies to maximize potential benefits and minimize adverse impacts; gathered and incorporated community feedback on recommendations	Recognitional, procedural, capabilities justice
Reporting	Documented HIA activities; communicated results to stakeholders and the public through materials, presentations, and meetings	Recognitional, procedural, capabilities justice
Evaluation and monitoring	Process evaluation determined if HIA met the objectives; planned for monitoring to determine effect on implementation and health outcomes	Recognitional, procedural, capabilities justice, distributional
